# The Effect of Various Ti6Al4V Powders on the Behavior of Particle-Reinforced Polyester Matrix Composites

**DOI:** 10.3390/polym15132904

**Published:** 2023-06-30

**Authors:** Erdoğan Teke, Elif Alyamaç Seydibeyoğlu, Mehmet Özgür Seydibeyoğlu

**Affiliations:** 1 Program of Materials Science and Engineering, Graduate School of Natural and Applied Science, Izmir Kâtip Celebi University, 35620 Izmir, Turkey; 2 Department of Petroleum and Natural Gas Engineering, Izmir Kâtip Çelebi University, 35620 Izmir, Turkey; 3 Department of Materials Science and Engineering, Izmir Kâtip Çelebi University, 35620 Izmir, Turkey

**Keywords:** polyester composite, Ti6Al4V powder, thermal conductivity, mechanical strength

## Abstract

In this study, recycled and commercial Ti6Al4V powder was added to polyester composites at various filling ratios by weight fraction. Three weight fractions of Ti6Al4V particles, 0%, 5%, and 10%, were chosen for study. By examining the mechanical and microstructural properties of polyester composites, the effects of the Ti6Al4V powder proportion by weight fraction and particle size parameters were investigated. With the filler loading, a 39% increase in the tensile strength of the composites was achieved. A minor decrease in flexural strength was observed at 5% filler weight fraction. The addition of the recycled Ti6Al4V powder to the polyester matrix slightly reduced the thermal conductivity of the composite over that of the neat polymer. However, the incorporation of the commercial Ti6Al4V powder fillers in the polyester matrix considerably increased the thermal conductivity of the composites, suggesting several potential uses. The presence of high levels of oxygen in the powder led to reduced thermal conductivity of the composites due to the reduction in phonon scattering.

## 1. Introduction

Composite materials first emerged in the middle of the 20th century and are now one of the most important areas of materials and manufacturing technology [1,2]. They are ideal for a variety of applications in the industrial sectors of aerospace, automotive, construction, sports, and bio-medicine, among many others, due to their excellent properties such as being lightweight and corrosion-resistant [3,4].

Modern technologies frequently require materials with very complex property combinations that are not achievable with more traditional metal alloys, ceramics, and polymeric materials. This is particularly true for materials used in transportation, undersea, and aircraft applications. For instance, structural materials with low density, strength, stiffness, resistance to abrasion and impact, and resistance to corrosion are increasingly sought after by aircraft engineers. The combination of these qualities is ideal in terms of physical strength. Strong materials are usually relatively thick, and increasing strength or stiffness typically causes a reduction in impact strength [5]. These materials have exceptional mechanical and structural qualities, including a high strength-to-weight ratio, resistance to fire, chemicals, corrosion, and wear, as well as being inexpensive to produce [6,7].

Composites are classified based on their morphology of reinforcement (fiber, particulate-reinforced, and laminate composites) or their matrix material (metal, ceramic, or polymer matrix composite). In a polymer composite material, the matrix is a resin and the reinforcement is in the form of dispersed particles, which act as the second phase. A wide range of microstructures can be obtained using combinations of matrix materials and reinforcement. The relationship between the properties and the structure of two-phase materials has been studied by many authors. In commercial production, low-cost particulate fillers are added to plastics for primarily economical reasons, as well as to improve molding characteristics. It is not only the material properties of the two components or the volume fraction of the filler which governs the deformation behavior, but also their shape, size, orientation, and the state of adhesion between the filler and the matrix [8].

Polyester resins and other thermosetting plastics are frequently utilized as matrix materials because they evenly distribute stresses in all directions and can withstand shocks and vibration. The most frequently utilized resin system is polyester resin, notably in the marine industry. The liquid mixtures of low molar mass reactants, such as monomers, which are used to make thermosetting plastics, polymerize to form strongly cross-linked network polymers [9,10,11]. The qualities of the particle filler dispersed in the composite are enhanced. Polymer composites manufactured in this manner have found numerous applications as lightweight, high-strength materials. Polymer matrix composites can be reinforced with fibers (synthetic or natural), whiskers, and particulate materials. Natural fillers include minerals such as calcium carbonate, mica, talc, and some agricultural byproducts of synthetic fillers, including processed mineral products such as carbon black, fumed silica, and aluminum hydroxide [12,13]. The sizes of particulate fillers range from 0.1 µm to approximately 2 mm. It can be inferred from the literature that the research on Ti6Al4V-powder-reinforced polymer composites is relatively limited [14].

In recent years, metal particles have been viewed as prospective reinforcing materials for various composites [15]. It is stated in the literature that many metal particles such as aluminum, copper, zinc, stainless steel, silver, and nickel increase the electrical and thermal conductivity of metal-filled polymer composites [16].

Yaman and Taga [17] investigated the thermal and electrical conductivity of copper-filled polyester composites. According to their study, both the thermal and electrical conductivity of polyester composites increase with increasing filler particle sizes and filler contents. As a result, the particle size distribution and content of fillers play significant roles in determining the thermal properties of composites. In another similar study, the electrical behavior of aluminum-powder-reinforced polyester composites was investigated. In the study by Berger and McCullough, an increase in electrical conductivity was achieved as the filler content increased [18].

Alam et al. added multi-walled carbon nanotubes (MWCNTs) to a polyester matrix at different compositions (0.1, 0.3, and 0.5 wt.%). They concluded that 0.3 wt.% MWCNT was the optimum amount of filler to mechanically and thermally improve the polyester matrix [19].

In another study, Krishnasamy et al. investigated the effect of aluminum and copper wire mesh on the thermal and mechanical properties of epoxy composites. It was concluded that the tensile strength of copper- and aluminum-reinforced epoxy composites increased [20].

With composites, it is possible to reduce the overall production cost by embedding waste metal particles from other manufacturing processes or recycled materials in a polymer.

This study concerns the evaluation of mechanical properties such as tensile and flexural strength for different weight ratios of Ti6Al4V-particulate-filled polyester composite. Titanium alloys, which are especially preferred in aerospace applications, exhibit tensile strength, creep and fatigue strength, and fracture toughness; they contain α and β stabilizing elements to achieve the necessary mechanical properties such as fatigue cracking, stress-abrasion cracking, and oxidation resistance. These properties classify Ti6Al4V as a precious metal. Recycling chip materials in different forms will provide both environmental and economic benefits. The recycling of Ti6Al4V chips generated during machining is limited due to difficulties in the recycling process. In this study, Ti6Al4V chips were converted into powder form using mechanical milling, and the potential use of the obtained Ti6Al4V powder as a reinforcement material in a polyester matrix was investigated. This article focuses on the effect of the addition of Ti6Al4V particles on the mechanical properties, and thermal stability of the obtained polyester composite.

## 2. Materials and Methods

### 2.1. Materials

#### 2.1.1. Preparation of Ti6Al4V Powder

Ti6Al4V scraps obtained in chip form following machining served as the starting materials. The machining chip was supplied by Metrosan Company in Manisa, Turkey. Ti6Al4V alloy powder was utilized as a reinforcement; it was recycling powder obtained from Ti6Al4V chips following mechanical milling. The milling process of the Ti6Al4V chip was performed using the Retsch RS200 vibratory disc mill. In the milling process of the Ti6Al4V chips, a milling duration of 60 min was selected and a milling speed of 1500 rpm was used. In this study, irregularly shaped recycled Ti6Al4V powder obtained as a result of the milling process was used. The characteristics of Ti6Al4V powder used as reinforcement are given in detail in Table 1.

**Figure 1:**
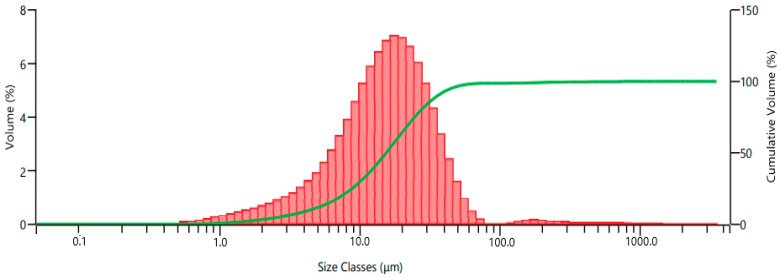
Particle size distribution of powder A.

**Figure 2:**
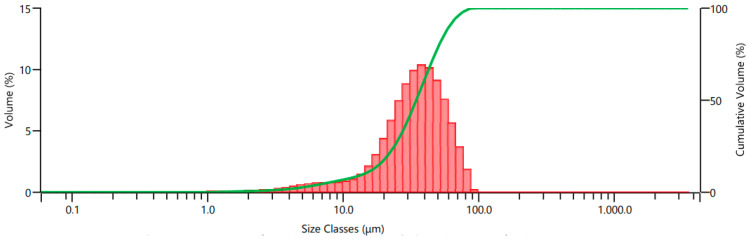
Particle size distribution of powder B.

Ti6Al4V powder obtained from the milling process was subjected to 325 and 625 mesh sieve analysis, and particle size classification was performed. Two types of recycled Ti6Al4V powder obtained after sieve analysis were coded as A and B according to their particle size distribution. The commercial Ti6Al4V powder was coded as C. The particle size distributions of the recycled powders, are dispersed by a range of dry units, were determined using a laser diffraction particle size measurement instrument (Malvern Panalytical Mastersizer 3000, Malvern, UK). Two kinds of recycled Ti6Al4V powder fillers, coded as A and B, were utilized, with mean particle sizes of 15.4 μm and 34.6 μm, respectively. Particle size distribution histograms of powders A and B are given in Figure 1 and Figure 2, respectively. Commercial Ti6Al4V powder with a particle size of 15–45 μm was also used for comparison in the same parameters. The commercial Ti6Al4V powder was obtained from the Nanokar Company (Istanbul, Turkey).

Table 1 details the oxygen concentration in the recycled A and B powders and the commercial Ti6Al4V powder. The oxygen ratios in the powders used in the study were measured using the LECO TC400 Nitrogen/Oxygen determinator, USA. The increase in oxygen content of recycled powder is due to the milling atmosphere and the increased particle surface area (particle size is decreased). Additionally, this situation is attributable to surface contamination of the starting chip and the jar’s temperature.

**Figure 3:**
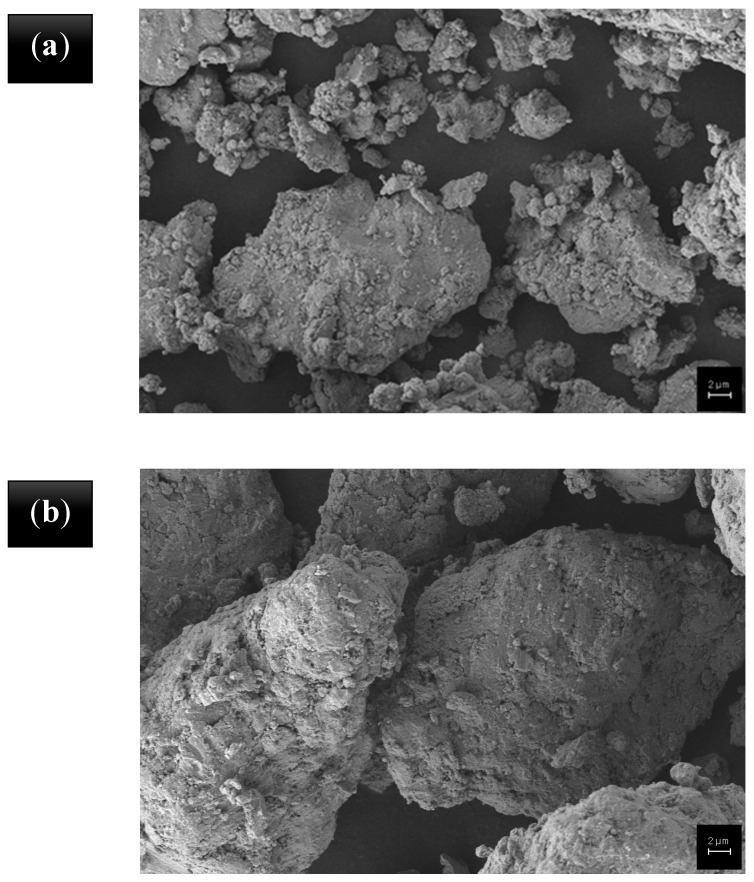

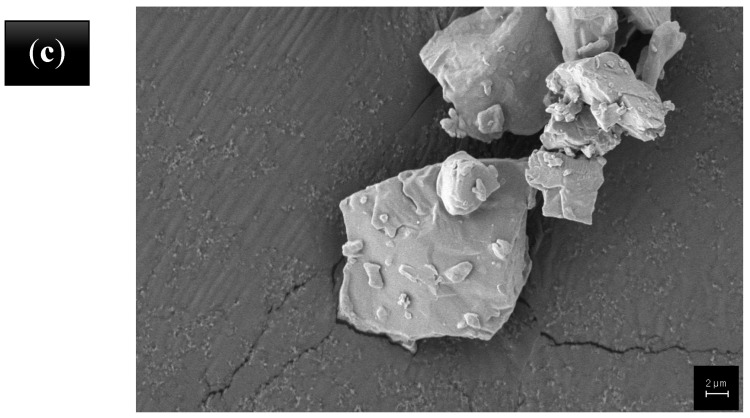
Particle morphology of powder A (**a**), powder B (**b**), and the commercial powder, C (**c**).

SEM images of the commercial and recycled Ti6Al4V powders are given in Figure 3. The recycled powders exhibit a rounded morphology and satellite nanoparticles on their particles. The shape of the commercial Ti6Al4V particles is angular and their morphology is smoother than that of recycled Ti6Al4V particles. It is known from the literature that the morphology of the particles has an effect on the mechanical and thermal properties.

#### 2.1.2. Polymer Matrix

A commercially available Polipol 353 casting-type polyester resin was used as the matrix material. This is a thermoset type of polymer matrix material. Polipol 353 casting-type polyester resin is an orthophthalic unsaturated polyester resin designed for general-purpose, standard casting applications. The polyester resin with added cobalt accelerator was used together with the hardener; both were purchased from Yücel Kompozit (Izmir, Turkey), as detailed in Table 2. Methyl Ethyl Ketone Peroxide (MEK-P) is used for curing general-purpose unsaturated polyesters at room temperature; it is generally added into a resin at a rate of 1% (by weight).

### 2.2. Experimental Methods

#### 2.2.1. Manufacturing of Composites

The polyester resin and MEK-P hardener (%1) were mixed according to the datasheet provided by the supplier. Three formulations, 0, 5, and 10 wt.% Ti6Al4V powder in polyester, were developed, as summarized in Table 3.

A specially designed and fabricated silicon mold was used for this purpose to avoid sticking during curing. The Ti6Al4V powder was oven-dried for 24 h at 80 °C. Ti6Al4V powder was mixed with polyester at a low speed by stirring mechanically to avoid bubbles, and the hardener was mixed into the Ti6Al4V powder/polyester resin mixture. For the synthesis of reinforced polyester composites, the mass ratio of polyester to reinforcement was modified to yield a total of 40 g.

In the case of the particulate-reinforced composites, Ti6Al4V powder was added to the polyester resin and mixed well in a continuous stirring process until a uniform mixture was observed; then, the hardener was added into the Ti6Al4V/polyester mixture. Stirring of the mixture continued for a certain duration depending on the exothermic reaction. The neat polyester resin samples were also prepared under similar processing conditions.

#### 2.2.2. Characterization of Composites

**Figure 4:**
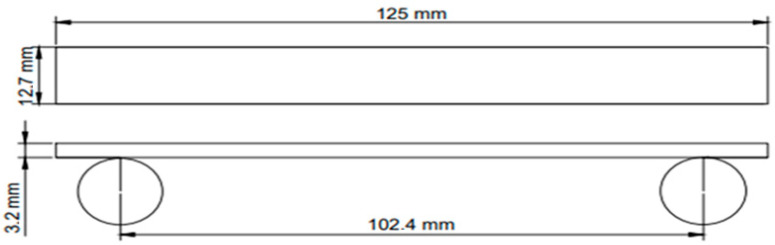
The dimensions of the three-point bending test specimens.

Five specimens of each type were tested. The results were averaged from five tests. The three-point bending test was performed with a constant loading speed of 1 mm/min at room temperature, and a span length of 102.4 mm, as per American Society for Testing and Materials (ASTM) D790, using a Shimadzu universal testing machine. The dimensions of the specimens were 125 mm × 12.7 mm × 3.2 mm with a span of 102.4 mm length, as given in Figure 4. A fixed span-to-depth ratio of 32 was used.

**Figure 5:**
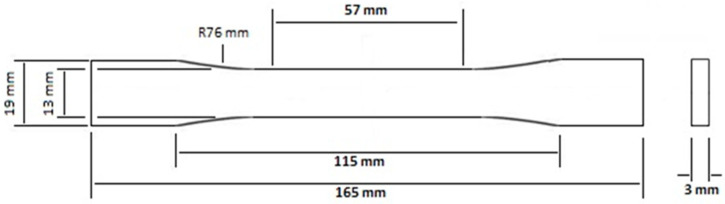
Tensile test specimen dimensions (ASTM D638-03 Type I).

Laboratory tests were performed using the Shimadzu universal tensile strength testing machine (AG-IC 100kN); loading speed of the deformation corresponded to 1 mm/min at room temperature. The tensile test specimen dimensions according to ASTM D638-03 Type I are given in Figure 5.

Thermal conductivity measurements were conducted using a C-Therm TCI test machine, in accordance with ASTM D 5470. The cylindrical testing samples had a diameter of 20 mm and a height of 3 mm. The thermal conductivity of various proportions of Ti6Al4V-filled polyester composites was determined.

Morphological investigations of the powders and composites were carried out using Carl Zeiss 300VP scanning electron microscopy (SEM) apparatus.

## 3. Results

All of the samples, neat and PA-PB-PC, for all cases of filler weight fractions and sample dimensions, were successfully produced with no visible defects or other issues. The standard deviation values of tensile and bending test results were high because each sample was produced by hand.

### 3.1. Tensile Test Results

**Figure 6:**
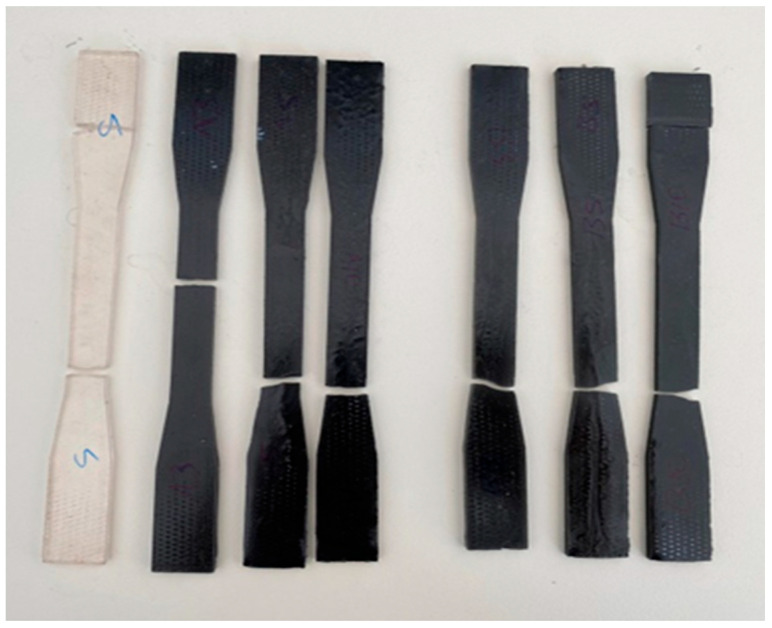
Fractured regions of the samples after the tensile test.

After the tensile strength analysis, the fractured regions are given in Figure 6. Moreover, Table 4 shows the effect of adding different wt.% and particle sizes of Ti6Al4V powder on the tensile strength of polyester composites.

The mechanical properties of particulate–polymer composites depend on the particle loading, particle size distribution, and particle–matrix interface adhesion [21]. The tensile strength of Ti6Al4V-powder-reinforced polyester composites, except for the PC5 composite, increased when compared with the neat polyester composite. It is clear that the tensile strength is significantly dependent on particle proportion.

The results from the Shimadzu (AG-IC 100 kN) machine and the Young’s modulus values of the composites are given in Table 4.

The surface area of the particles increased as the particle size decreased. Therefore, the PA10 composite, which had the largest particle surface area, had higher tensile strength. In addition, PA and PB composites had higher tensile strength compared with PC composites. The morphologies of particles A and B were more irregular than particle C. The irregular form of the particles provided good correlation, such as interlocking between the polymer matrix and the filler.

**Figure 7:**
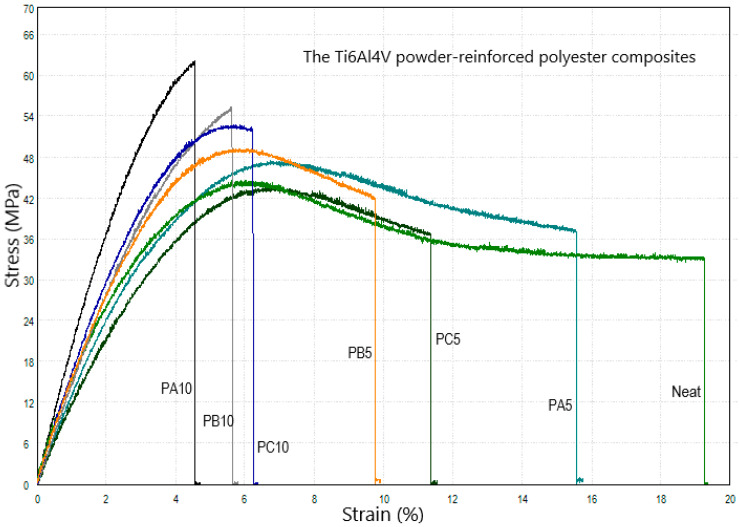
Tensile stress–strain curves of the ‘Ti6Al4V-powder-reinforced’ polyester composites.

In Figure 7, the polyester composites show decreasing strain (ductility) as the Ti6Al4V concentration increases. The lowest strain values were obtained with the PA10 sample. The proportion of filled particles in polymer matrix composites (PMCs) was significant for the general mechanical behavior of the composite. On the other hand, extreme particle adding can easily cause agglomeration of the fillers, therefore introducing defects to the matrix [22]. The tensile strength increased slightly when 5% by weight of the particles was added, while the tensile strength increased significantly when 10% by weight of the particles was added to polyester resin. An improvement in tensile strength as the particle proportion increased, indicated strong interfacial bonding between the particle and the matrix. The particle size distribution was also a parameter that affected the mechanical properties of the filled matrix [23].

The tensile strength also increased as the particle size decreased for the same weight fraction of the fillers due to the increased interfacial area between the particle surface and the polymer matrix. The commercial Ti6Al4V powder was used as reinforcement in PC5 and PC10 composites produced with the same parameters. The tensile strength of the commercial Ti6Al4V-powder-reinforced composites is lower than that of the recycled Ti6Al4V-powder-reinforced polyester composites. It is known that the particle shape affects the mechanical behavior in PMCs [21]. The commercial Ti6Al4V powder had a more regular morphology than the recycled Ti6Al4V powder. The irregularly shaped reinforcement connected with the matrix in an interlocking manner [24].

### 3.2. Three-Point Bending Test Results

The flexural strength of a particle-reinforced polymer matrix composite is affected by factors such as the bonding strength between the particles and the matrix, particle size distribution, and particle loading. The bending test is a simple method for testing supports that involves a three-point bending test, in which a loading pin is lowered from above at a constant rate. The specimen is placed on two supporting pins at a set distance from the specimen, and the fixture is mounted on a universal testing machine at crosshead speed and load was gradually applied until breakage of the specimen occurred. The test results are affected by details of the test preparation, conditioning, and load rate. An average of five specimens was taken. Table 5 shows the effect of adding Ti6Al4V powder with different weight fractions on the flexural strength and flexural modulus of polyester composites.

The results indicated that the flexural strength decreased when the particle content was 5 wt.% and then increased with the particle content increasing up to 10 wt.%. For the PA5-PB5-PC5 composites with a reinforcement ratio of 5 wt.%, the decreased flexural strength led to a reduction in bending stress. This may be a result of poor bonding of the particles with the matrix and the inhomogeneous filling of reinforcement in matrices (poor particle distribution) that diminished the support to bending stress. The particles easily detached when the composite was subjected to load, which could lead to the formation of large voids [25].

The flexural strength values of the PA10-PB10-PC10 composites were significantly enhanced compared with the unfilled polyester composite due to good particle dispersion and strong polymer/filler interface adhesion for effective stress transfer [26,27]. This implies that the particle proportion has a significant effect on composite strength. For the particulate composites, the flexural strength depends on the transfer of stress between the matrix and particles [28]. The greatest improvement in flexural strength was obtained at a 10 wt.% content of PB10. The lowest flexural strength of the composites was obtained with a 10% particle proportion in the PC10 composite.

### 3.3. Thermal Conductivity Test Results

The experimental results of the thermal conductivity are presented in Table 6. The effective thermal conductivity of a composite material composed of one type of filler introduced into a polymer matrix depends on the thermal conductivity of the components, the filler’s shape, size, concentration, their dispersion into the polymer, and the thermal interfacial resistance [29].

The addition of the recycled Ti6Al4V powder to the polyester matrix slightly reduced the thermal conductivity of the composite over that of the neat polymer. However, the incorporation of the commercial Ti6Al4V powder filler in the polyester matrix significantly increased the thermal conductivity of the PC5-PC10 composites. In the previous sections, the properties of Ti6Al4V powders used as fillers were detailed. The morphology of the commercial Ti6Al4V powder was more regular compared with that of the recycled Ti6Al4V powder. Thus, the Ti6Al4V filler particle shape and the distribution of filler particles are the critical factors impacting the thermal properties of metal-filled composites. The result is most likely due to the different particle shapes between the commercial and recycled types of filler, since the particle shape has a substantial effect on thermal conductivity [30]. The presence of oxygen in the A and B powders used as fillers acts as a vacancy and negatively affects the thermal conductivity. Therefore, the decrease in the thermal conductivity of PA and PB can be attributed to the high oxygen content. However, the incorporation of C powder fillers with a lower oxygen content in the polyester matrix significantly increased the thermal conductivity of the composites.

### 3.4. Morphology of the Composites

**Figure 8:**
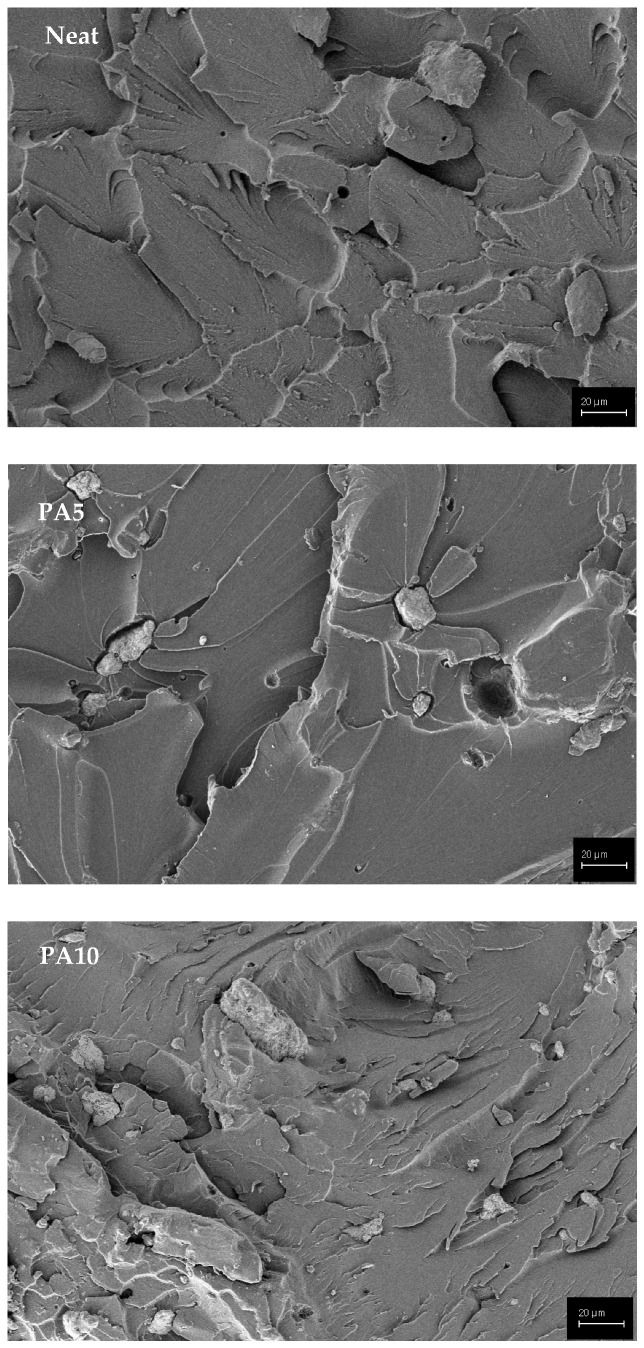

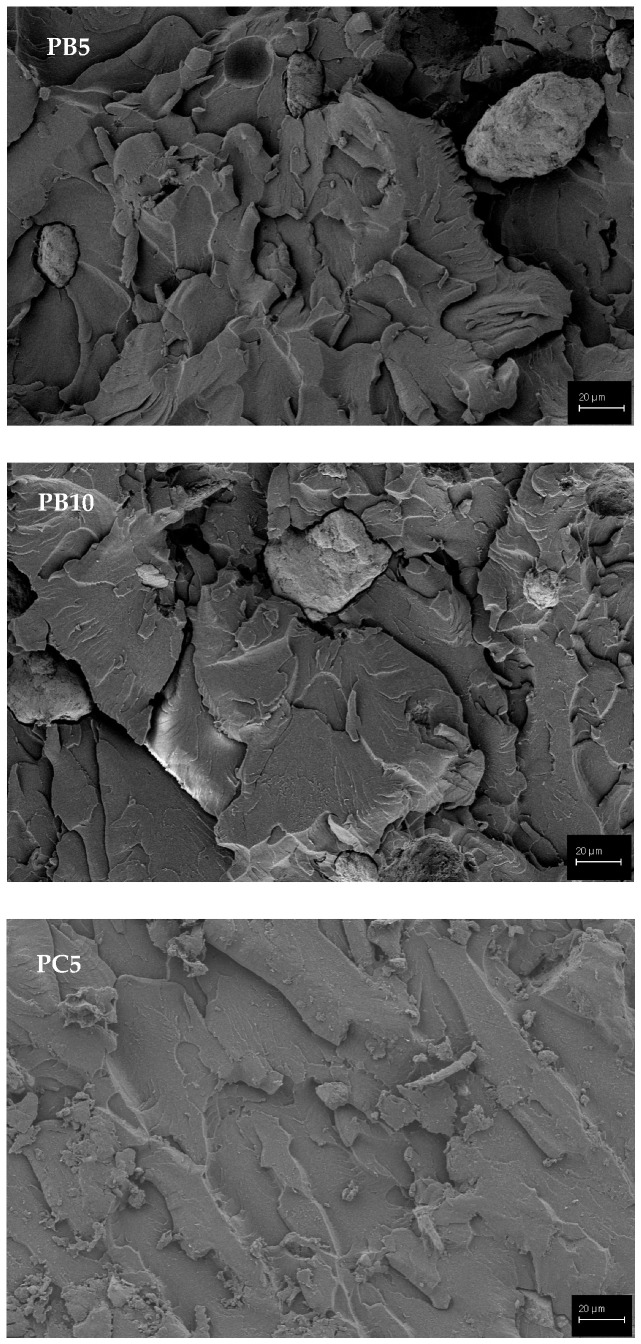

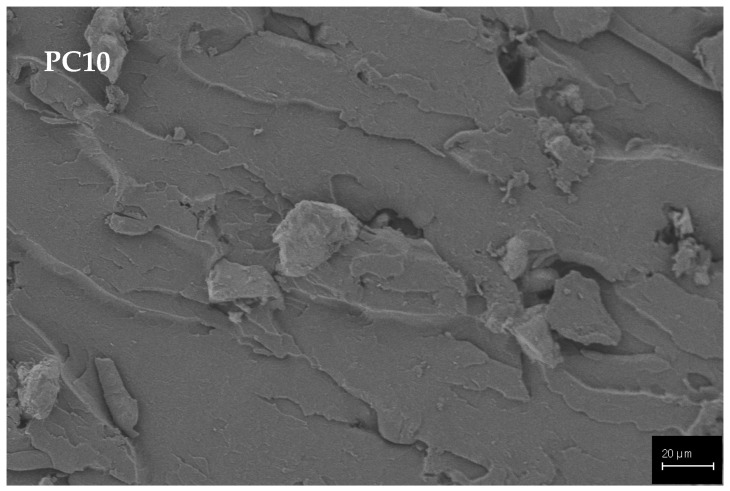
Morphology of the polyester composite specimens at different magnifications.

The fracture surfaces of neat polyester and the Ti6Al4V-particle-reinforced composite samples were investigated for various fracture features under a scanning electron microscope. According to Figure 8, the results of the experiment also show the irregular stratification of filler micro particles in the matrix. According to this observation, the quality of powder-matrix mixing process was not sufficient and better mixing may improve the powder dispersion in the matrix. The brittle-type fracture mode was observed in all polyester composite samples. However, micron-sized voids formed by the particles were observed on the fracture surfaces. During the tensile test, cracks formed at the applied stress. The lines around the particles indicated that it caused the extra stress concentration. When adding to the particles to the matrix, the wetting of the particles with the matrix was very important [31]. The presence of particle traces on the fracture surfaces indicated that the particles were held in place by the matrix material.

### 3.5. Effect of Powder Oxygen Concentration on the Mechanical and Thermal Properties of Composites

We focused on the effects of oxygen on the mechanical properties in the polyester composites. The measured oxygen concentrations in the A, B, and commercial Ti6Al4V powder were 34,840 ppm, 20,966 ppm, and 2565 ppm, respectively. The results show that the powder oxygen concentration had a significant effect on the mechanical properties of the Ti6Al4V-powder-reinforced polyester composites. The tensile and flexural strength of PC samples decreased compared with PA-PB samples with high oxygen concentrations. High oxidation may contribute to achieving strong particle–matrix adhesive bonding. In general, high oxide concentrations can provide strong particle–matrix interfacial bonding [32,33]. The oxidation level of the particles used as filler also affects the thermal properties of the composites. Although a high oxygen content improves the mechanical properties, it has a negative effect on thermal conductivity. Thermal conductivity decreased in PA and PB composites due to the high oxygen contents of powders A and B. However, a significant increase in thermal conductivity was observed in PC samples with low oxygen contents. The presence of high oxygen reduced thermal conductivity due to the reduction in phonon scattering. Phonon scattering from grain boundaries and internal defects are significant factors affecting thermal conductivity. The presence of high oxygen reduced thermal conductivity due to reduced phonon scattering. As a result, the thermal conductivity determined at high oxidation levels was lower than that measured at low oxidation levels [34,35,36].

## 4. Conclusions

Circular economy and recycling studies are critical in today’s materials world. In addition to recycling, upcycling processes that convert waste streams to value-added products are a vital subject of study for humanity and the environment.

In this study, we have investigated another waste product obtained from the medical industry. The novelty of this work is that the effect of the recycled and commercial Ti6Al4V powder on the properties of the polyester composite was studied. In this study, the effects of particle size, shape, and filler content on the mechanical behavior of polymer matrix composites reinforced with the recycled and commercial Ti6Al4V powder were investigated.

With the filler loading, an increase in the tensile strength was achieved due to the increased surface contact area between the particles and the resin. A small decrease in bending strength with a filler weight fraction of 5% was observed. This may be because a 5% amount of filler is not sufficient to transmit the stress transfer from the matrix to the filler. However, an increase in flexural strength was observed with a 10% filler weight fraction. The addition of the recycled Ti6Al4V powder to the polyester matrix slightly reduced the thermal conductivity of the composite compared with that of the neat polymer.

## Figures and Tables

**Table 1:** The oxygen concentration in the recycled A and B powders and the commercial Ti6Al4V powder.

Powder	d(10) μm	d(50) μm	d(90) μm	Oxygen (ppm)
A	4.4	15.4	35.7	34,840
B	14.4	34.6	60.7	20,966
C (Commercial)	-	15–45	-	2565

**Table 2:** The unsaturated polyester specifications.

Appearance	Transparent
Color	Clear
Density	1.11 g/cm³
Flash point	32 °C
MEK-P hardener	% 1
Usable life	30 min/25 °C
Cure time	12 h/25 °C

**Table 3:** The composition ratio of Ti6Al4V powder in the polyester composite.

Sample Code	Polyester (wt.%)	Ti6Al4V Powder (wt.%)	Powder Type
Neat	100	0	-
PA5	95	5	Recycled
PA10	90	10	Recycled
PB5	95	5	Recycled
PB10	90	10	Recycled
PC5	95	5	Commercial
PC10	90	10	Commercial

**Table 4:** Tensile strength of polyester reinforced with different wt.% of Ti6Al4V powder.

Sample	Tensile Strength (MPa)	Young’s Modulus (GPa)
Neat	44.83 ± 2.46	1.21 ± 0.03
PA5	47.42 ± 4.20	1.17 ± 0.13
PA10	62.17 ± 3.16	1.92 ± 0.10
PB5	49.35 ± 4.02	1.35 ± 0.13
PB10	55.38 ± 3.34	1.39 ± 0.09
PC5	43.58 ± 2.22	0.99 ± 0.08
PC10	52.67 ± 2.71	1.48 ± 0.05

**Table 5:** Flexural strength of the polyester composites.

Sample	Flexural Strength (MPa)	Flexural Modulus (MPa)
Neat	106.61 ± 2.44	2.280 ± 0.05
PA5	103.92 ± 4.58	2.525 ± 0.11
PA10	118.33 ± 3.71	2.945 ± 0.09
PB5	101.78 ± 4.40	1.973 ± 0.10
PB10	135.85 ± 3.03	3.098 ± 0.08
PC5	93.54 ± 3.82	1.956 ± 0.08
PC10	104.09 ± 2.87	2.774 ± 0.06

**Table 6:** The experimental results of the thermal conductivity measurements.

Sample	Thermal Conductivity (W/mK)
Neat	0.2502 ± 0.001
PA5	0.2271 ± 0.003
PA10	0.2317 ± 0.003
PB5	0.2121 ± 0.001
PB10	0.2344 ± 0.003
PC5	0.6079 ± 0.020
PC10	0.6319 ± 0.010

## Data Availability

All the data is presented are new. This article is based on Dr. Erdogan Teke’s PhD thesis that was finished in March 2023.
